# Improvement of domain-level ortholog clustering by optimizing domain-specific sum-of-pairs score

**DOI:** 10.1186/1471-2105-15-148

**Published:** 2014-05-18

**Authors:** Hirokazu Chiba, Ikuo Uchiyama

**Affiliations:** 1 National Institute for Basic Biology, National Institutes of Natural Sciences, Nishigonaka 38, Myodaiji, Okazaki 444-8585, Japan

**Keywords:** Ortholog group, Domain, Multiple alignment, Sum-of-pairs score

## Abstract

**Background:**

Identification of ortholog groups is a crucial step in comparative analysis of multiple genomes. Although several computational methods have been developed to create ortholog groups, most of those methods do not evaluate orthology at the sub-gene level. In our method for domain-level ortholog clustering, DomClust, proteins are split into domains on the basis of alignment boundaries identified by all-against-all pairwise comparison, but it often fails to determine appropriate boundaries.

**Results:**

We developed a method to improve domain-level ortholog classification using multiple alignment information. This method is based on a scoring scheme, the domain-specific sum-of-pairs (DSP) score, which evaluates ortholog clustering results at the domain level as the sum total of domain-level alignment scores. We developed a refinement pipeline to improve domain-level clustering, DomRefine, by optimizing the DSP score. We applied DomRefine to domain-level ortholog groups created by DomClust using a dataset obtained from the Microbial Genome Database for Comparative Analysis (MBGD), and evaluated the results using COG clusters and TIGRFAMs models as the reference data. Thus, we observed that the agreement between the resulting classification and the classifications in the reference databases is improved at almost every step in the refinement pipeline. Moreover, the refined classification showed better agreement than the classifications in the eggNOG databases when TIGRFAMs was used as the reference database.

**Conclusions:**

DomRefine is a useful tool for improving the quality of domain-level ortholog classification among microbial genomes. Combining with a rapid domain-level ortholog clustering method, such as DomClust, it can be used to create a high-quality ortholog database that can serve as a solid basis for various comparative genome analyses.

## Background

Identification of orthologs constitutes the basis for comparative analysis of multiple genomes. It provides not only a foundation for inferring the evolutionary history of genes and genomes but also an important clue for inferring protein functions [1]. Originally, orthologs were defined as a pair of genes diverged from the same ancestral gene by speciation, whereas paralogs are a pair of genes diverged by gene duplication [2]. Because the functions of orthologs are typically more conserved than those of paralogs, orthology relationships are often used to transfer functional annotations between organisms [3,4]. The concept of orthology has been extended from pairs of organisms to multiple organisms by clustering orthologs into ortholog groups [5]. Ortholog groups are a vital resource for comparative analysis of multiple genomes and provide a basis for phylogenetic profile (the presence and absence patterns of genes in genomes) analysis [6].

Owing to rapid progress in sequencing technologies, an increasing number of genomes have been sequenced. In particular, accumulation of microbial genome data is remarkable [7]; several thousand genomes across diverse taxa have already been sequenced, and even more data have been generated as metagenomes from various environmental samples. A reliable method for identifying ortholog groups among multiple genomes is needed for comparative analysis of this huge amount of microbial data. In prokaryotes, the prevalence of horizontal gene transfers (HGTs) makes accurate ortholog inference infeasible [8]. Therefore, a relaxed condition, i.e., closest homologs in different species regardless of HGTs, is usually used as an alternative definition of orthology for prokaryotic genome comparison [4].

Several previous studies have developed orthology inference algorithms and ortholog databases [9,10]. One of the most basic algorithms to identify orthologs is the bidirectional best hit (BBH) approach for a pair of species [11]. The BBH approach was extended to deal with multiple species by applying clustering methods to the graph of BBH relationships; this approach for creating ortholog groups is known as a graph-based method [5,12-15]. The Clusters of Orthologous Groups (COGs) database is a pioneering study of graph-based methods and is still one of the most popular ortholog databases, although it is no longer updated [5,12]. The eggNOG database was later constructed by extending COGs incrementally using a computational method [13]. Another approach for creating ortholog groups is based on the phylogenetic tree of genes and is called a tree-based method. Such a method produces more reliable results than graph-based methods but at the expense of higher computational costs [16-19]. The DomClust algorithm [20], which is used to create ortholog groups in the Microbial Genome Database for Comparative Analysis (MBGD) [21], adopts an intermediate approach, where ortholog groups are identified on the basis of hierarchical clustering trees created from a graph of all-against-all pairwise similarity relationships.

**Figure 1:**
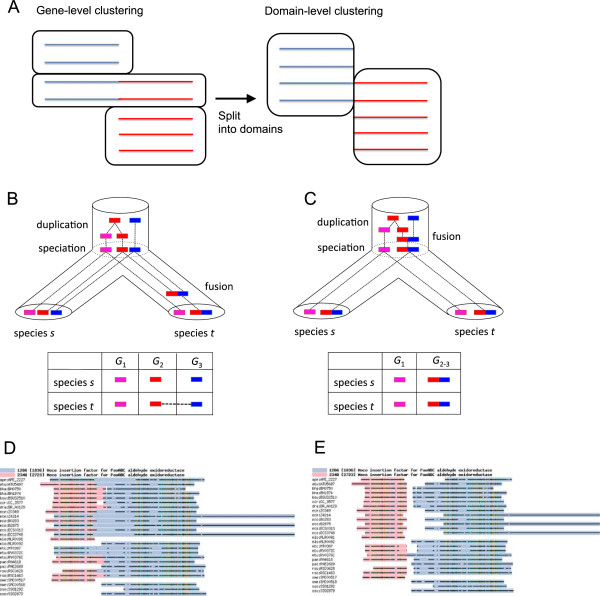
**The concept and examples of domain-level ortholog clustering. (A)** Schematic illustration of ortholog clusters containing fusion proteins. The lines represent protein sequences, and red and blue colors represent two distinct domains of the proteins. **(B, C)** Groups of orthologous domains in two evolutionary scenarios: the case of gene fusion after speciation **(B)** and gene fusion before speciation **(C). (D, E)** Appropriate re-splitting of proteins refines the domain-level ortholog clustering. Examples of inconsistent domain boundaries **(D)** and a refined version of the boundaries **(E)** are shown in multiple alignments, where two adjacent domains are colored in light blue and pink, respectively (see Methods for details of the alignment visualization tool).

Among numerous methods proposed to create ortholog groups, only a few methods consider orthology relationships at the sub-gene level. Figure 1A is a schematic illustration of ortholog clustering at the domain level, where fusion proteins comprising originally distinct proteins are included. With a simple clustering method that does not consider sub-gene level classification, a fused protein will be assigned to exclusively one of the clusters (Figure 1A, left). However, considering that each domain in the fused protein can have a distinct function that is shared among the corresponding orthologs, a natural method of grouping them is to split the fused proteins into domains and treat them separately (Figure 1A, right). Such a clustering procedure, called domain-level ortholog clustering, is a challenging problem because not only the cluster members but also the set of fusion proteins and domain boundaries within them must be identified. Some methods such as HOPS [22] use information of known domains such as those included in the Pfam database to identify domains and then identify orthologs within each domain. However, such approaches are unsuitable for comprehensive ortholog classification of the entire set of proteins because of their dependency on the existing domain database.

The orthologous domains considered here are orthologous gene subsequences that have been stable (unsplit) during evolution after speciation from a common ancestor. To clarify the difference between orthologous domains and conventional homologous domains, let us consider the following evolutionary scenarios (Figure 1B, C). In Figure 1B, a gene fusion event occurred after speciation. In this case, the fused gene is split into two subsequences in the orthologous domain classification. In Figure 1C, a gene fusion event occurred before speciation. In this case, full-length fused genes are classified in the orthologous domain group because the fused form is stable after speciation. In either scenario, there are two homologous domain groups: one is the blue domain and the other includes both the red and pink domains that are paralogous to each other. These examples illustrate that orthologous domains can be longer than homologous domains if domain reorganization occurs before speciation.

Note that the full length of a gene can be an orthologous domain. If the domain-reorganization event after speciation is either gene fusion or gene fission, the orthologous domain should correspond to the full length of a gene in at least one of the species (Figure 1B). Thus, the orthologous domain defined here is a suitable unit for functional annotation in comparative genomics, with gene fusion/fission events taken into consideration and seems well consistent with manually curated ortholog databases such as COGs, although there are no clear-cut criteria for splitting genes into subsequences in the COG construction procedure [23]. DomClust automatically detects a domain-reorganization event and splits a cluster into orthologous domains during the process of hierarchical clustering [20].

In practical applications, the determination of orthologous domains becomes more complicated because of several factors, including insertions/deletions of promiscuous domains and random disruption of coding sequences due to loss of function. These factors fragment orthologous domains into smaller pieces than expected as a unit of functional annotation. To avoid this over-splitting problem, the DomClust algorithm tries to split genes into the minimum number of domains required for ortholog clustering, i.e., a gene is split only when a different set of genes is putatively orthologous to each split segment with sufficiently large scores [20]. Moreover, DomClust merges two adjacent domains in its final step when genes in the fission form are much fewer than those in the fusion form [20]. However, such approaches do not always work well. Figure 1D illustrates a simple but typical example, where domain boundaries determined by DomClust are inconsistent in a multiple sequence alignment. Such inconsistent alignment boundaries are problematic because they not only cause incorrect sequence grouping but also lead to failure of the above mechanisms of DomClust to avoid over-splitting. This problem arises presumably because DomClust determines the boundaries using pairwise, rather than multiple, sequence alignments. Thus, utilizing multiple alignment information supposedly improves the accuracy of domain-level ortholog clustering (Figure 1E).

In this study, we present a method for improving domain-level ortholog classification using multiple alignment information. We designed a scoring scheme to evaluate the inferred domain organization on the basis of multiple alignments and developed procedures to improve the inference by optimizing the score. The improvement procedures included the merge of adjacent domains to fix the over-splitting problem and determination of optimal domain boundaries. In addition, a phylogenetic tree was created for each cluster to check the cluster members in terms of orthology relation. To evaluate the improvements, we compared the obtained ortholog groups with the original ones by examining the agreement with COG and TIGRFAMs, which are the manually curated reference databases.

## Results

### Overview of the method to improve domain-level ortholog clustering

**Figure 2:**
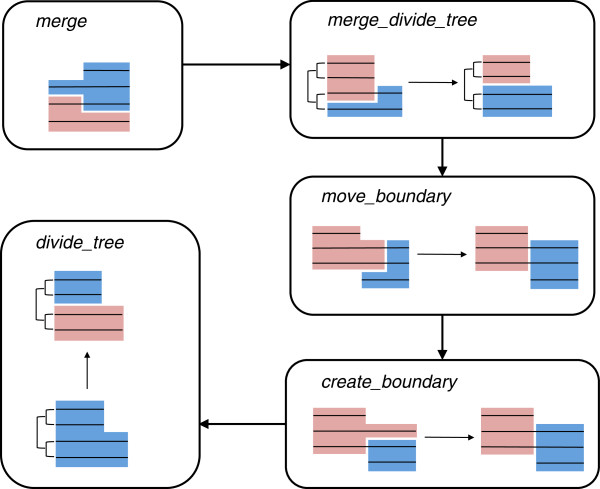
**The DomRefine pipeline.** The pipeline is given a domain-level ortholog clustering result and modifies domain organizations using five procedures. Domain organizations are illustrated using light blue and pink colors. Multiple alignments of amino acid sequences are represented by sets of aligned horizontal lines. Adjacent clusters are merged if the score increases by merging the clusters (*merge*). Given a pair of adjacent clusters, adjacent domains are temporarily merged and then divided into clusters considering score changes on the phylogenetic tree (*merge_divide_tree*). Existing boundaries are moved (*move_boundary*), and new boundaries are created (*create_boundary*). When species overlap between sub-clusters on the phylogenetic tree is detected, the cluster is divided into subgroups (*divide_tree*).

In this study, we assumed DomClust results as the input to our method, although any other domain-level clustering could have been applied. As illustrated in Figure 1A, a split of a protein sequence during domain-level ortholog clustering leads to the creation of adjacent domains that belong to different clusters (adjacent clusters). Pairs of adjacent clusters were the targets of our refinement procedure. For each pair of adjacent clusters in the input, a multiple alignment of protein sequences contained in either cluster was created and used in our refinement procedure. A domain-specific sum-of-pairs (DSP) score was introduced to evaluate the domain organization. The DSP score is based on the sum-of-pairs (SP) score [24]. However, it is calculated for each domain and inconsistencies in domain boundaries are evaluated as gaps so that the sum of the DSP scores in the alignments of adjacent clusters reflects the quality of domain classification. We defined five basic operations to modify and improve the domain organization by maximizing the DSP score and compiled them as a pipeline named DomRefine (Figure 2, see Methods for details). The first two procedures in the pipeline (*merge* and *merge_divide_tree*) were designed to solve the over-splitting problem; *merge* determined whether two adjacent clusters should be merged, whereas *merge_divide_tree* temporarily merged the adjacent clusters and then divided them into two groups (rather than split into two domains). The next two procedures (*move_boundary* and *create_boundary*) determined the optimized boundaries between the domains: the *move_boundary* procedure moved existing domain boundaries, whereas the *create_boundary* procedure introduced new boundaries. All the four procedures improved the domain organization on the basis of the maximization of the DSP score. In contrast, the last procedure (*divide_tree*) is a type of conventional tree-based approach for ortholog classification; it divided a cluster into subgroups along with the phylogenetic tree if the subgroups shared intraspecies paralogs.

**Figure 3:**
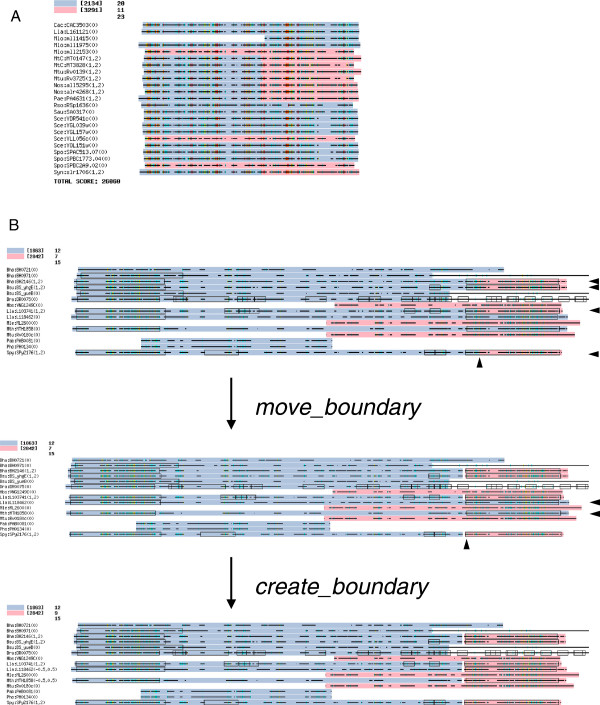
**Examples of improvement in domain-level ortholog clusters.** Examples of improvement by *merge***(A)** and *move_boundary* and *create_boundary***(B)** procedures are shown with multiple alignments, where two adjacent domains are colored in light blue and pink, respectively. The arrowheads indicate the domain boundaries to be modified. The black rectangles represent the matches of the TIGRFAMs models.

Figure 3 illustrates the examples of improved domain organization obtained by DomRefine. In the original classification by DomClust (Figure 3A), several proteins are split into domains, but the splitting pattern is inconsistent in the multiple alignment. In this case, canceling those splits to merge two clusters seemed to produce better classification. Indeed, the *merge* procedure merged these clusters because of the increase in the DSP score after merge, which resulted from the gain of the SP score between the newly aligned residues in the merged alignment and the disappearance of gaps owing to inconsistent domain boundaries. Figure 3B illustrates another example where the inconsistent domain boundaries were modified to lie at more appropriate positions. As a reference, the regions determined by the TIGRFAMs models are also illustrated. In the original classification, some proteins are split into domains, but the resulting domain boundaries did not coincide with the region detected by TIGRFAMs models. In addition, two proteins that also matched the same TIGRFAMs model are not split in the original classification. The *move_boundary* procedure moved all the existing boundaries at the same time in the multiple alignment to the best position on the basis of the DSP score. The subsequent *create_boundary* procedure created new boundaries, and the creation of these boundaries increased the DSP score. As a result of these procedures, we obtained domain boundaries that perfectly matched the region detected by TIGRFAMs models (Figure 3B).

### Overview of the results of domain-level ortholog clustering

Our method was tested on proteome sets retrieved from the COG and MBGD databases. The protein sequences from the COG03 dataset (including 66 organisms) were clustered into ortholog groups by our method, and the results were compared with the manually curated COG clusters for evaluation. To test the utility of our method in a more practical situation, we also constructed a larger dataset (the FAMILY dataset including 309 organisms) by selecting a representative organism from each taxonomic family of the MBGD database. For each of the COG03 and FAMILY datasets, we first applied DomClust to classify genes into ortholog groups and then applied the DomRefine pipeline to improve the classification. For the FAMILY dataset, we compared our results with eggNOG, which was constructed by computationally extending COG. In the comparison with eggNOG, we extracted the common proteome between FAMILY and eggNOG (FAMILY210 dataset including 210 organisms).

Table 1 summarizes the statistics of the ortholog clustering results. Although DomRefine had limited effects on the total number of clusters [from 7503 to 7307 (97.4%) for COG03; from 60775 to 57644 (94.8%) for FAMILY210], it caused significant changes in the number of split clusters. For the COG03 dataset, the number of split clusters produced by DomClust alone was higher than that in the original COG, reflecting the over-splitting problem of DomClust. After DomRefine was applied, however, the number of split clusters decreased drastically [from 2439 to 1562 (64.0%)] to approximately the same number as COG. This result was in line with expectations, given that DomRefine was designed to fix over-splitting problems. Similarly, in the FAMILY210 dataset, the number of split clusters was decreased from 15879 to 10942 (68.9%). In contrast, the number of split clusters in eggNOG was remarkably small (2333, which is only 3.6% of the total number of clusters) compared with the number in COG, DomClust, and DomRefine (range, 19%–33%). In particular, the number of split clusters in eggNOG is considerably lower than that in COG, on which it is based, presumably because of the lack of a procedure for splitting clusters into domains when creating new clusters not included in COG, i.e., non-supervised orthologous groups (NOGs) during the construction of eggNOG.

**Table 1:** Statistics of domain-level ortholog clustering results

**COG03 dataset**			**FAMILY210 dataset**		
**Method**	**No. of clusters**		**Method**	**No. of clusters**	
	***N***_***clust***_	***N***_***clust***_^***split***^		***N***_***clust***_	***N***_***clust***_^***split***^
COG	4814	1389 (29%)	eggNOG	64983	2333 (3.6%)
DomClust	7503	2439 (33%)	DomClust	60775	15879 (26%)
DomRefine	7308	1562 (21%)	DomRefine	57644	10942 (19%)

*N*_*clust*_ denotes the total number of clusters. *N*_*clust*_^*split*^ denotes the number of clusters that include proteins split into domains. The ratio of split clusters to the total number of clusters is shown in parenthesis.

**Figure 4:**
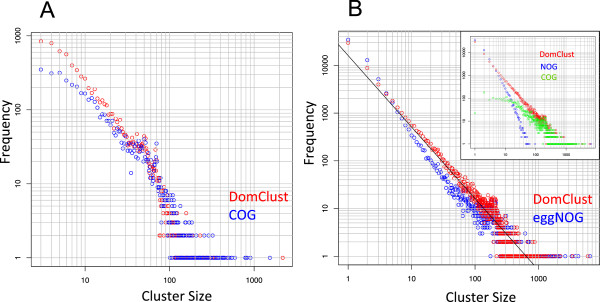
**Cluster size distributions of domain-level ortholog clusters.** Clustering results for the COG03 dataset **(A)** and the FAMILY210 dataset **(B)**. The red circles represent DomClust results. The blue circles represent COG data in **(A)** and eggNOG in the main plot area of **(B)**. In the upper right window of **(B)**, the eggNOG distribution is divided into COG-derived clusters (green circles) and NOG clusters (blue circles). The line represents log_10_*y* = −1.499 log_10_*x* + 4.206, obtained by the linear regression of the DomClust distribution on the log–log plot **(B)**.

For more detail, we also examined the distribution of the cluster size (the number of proteins in each cluster) (Figure 4). In general, the distributions of the cluster size show a near-linear relationship on a log–log plot, indicating that cluster sizes approximately follow a power-law distribution. For the COG03 dataset, the distributions of COG and DomClust show similar trends: the distributions deviate downward from the linear relationship at cluster sizes lower than 10 (Figure 4A) as observed previously [25]. This is because they retain only ortholog groups that have more than three members from (not closely related) different species (for results with smaller groups, see Additional file 1: Figure S1A). However, this trend is considerably prominent in COG than in DomClust, probably reflecting the feature of the COG classification that ortholog groups often contain small paralog groups that should be separated according to a rigorous definition of orthology.

For the FAMILY dataset, the DomClust distribution follows a linear relationship in the log–log plot (log_10_*y* = −1.499 log_10_*x* + 4.206, *R*^2^ = 0.90, Figure 4B), whereas the eggNOG distribution deviates from a linear relationship (for the fitted line, see Additional file 1: Figure S1B). When the eggNOG clusters are separated into COG-derived clusters and NOG, their distributions are substantially different (Figure 4B, upper right). The COG-derived cluster exhibits a curved distribution, deviating downward from the linear relationship at cluster sizes lower than 100. The NOG distribution has a steeper negative slope than DomClust (for the fitted line, see Additional file 1: Figure S1B) and deviates downward at cluster sizes greater than 10. In summary, DomClust, a fully automated clustering method, exhibited a power-law distribution in cluster size, whereas eggNOG, a combined approach of manual and automated methods, produced two different types of clusters and thus exhibited a relatively skewed size distribution.

### Assessment of the refinement procedures through the COG reconstruction test

**Figure 5:**
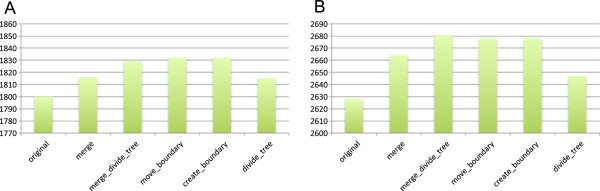
**Consistencies of the resulting clusters with COG clusters. (A)** COG02 dataset and **(B)** COG03 dataset. Vertical axes represent the numbers of one-to-one relationships.

To assess our refinement method, we examined whether our fully automated procedures could recover the manually curated COG database (COG02 including 43 organisms and COG03 including 66 organisms). To quantify the agreement of the clustering results between two methods (ours and COG) at the domain level, we first identified corresponding clusters as cluster pairs sharing at least one overlapping domain of the same protein and then extracted only those cluster pairs that had one-to-one correspondence (see Methods for details). The number of one-to-one corresponding cluster pairs against COG (*N*_*COG*_^*1to1*^) was then used as an indication for the agreement between two clustering results. Figure 5 presents the changes in *N*_*COG*_^*1to1*^ during the DomRefine procedures. We observed an increase in the agreement with COG during the *merge* and *merge_divide_tree* procedures (Figure 5A, B). These procedures exhibited greater changes than the subsequent procedures to modify boundaries (*move_boundary* and *create_boundary*). This is probably because increasing one-to-one relationships by moving a boundary requires exact matches of boundary positions; thus, $N{C_{\mathrm{COG}}}^{1\mathrm{to}1}$ is not a sensitive measure for capturing a moderate improvement in boundary positions. On the other hand, the consistency with COGs was decreased in the last procedure, *divide_tree*, which divides a cluster into subgroups to separate paralogs rather than modifying the domain organization. However, this result does not necessarily mean that *divide_tree* failed to improve ortholog classification, considering that a COG cluster often includes obvious outparalogs as members, resulting in a larger cluster than that produced by more rigorous ortholog grouping (see Discussion).

**Figure 6:**
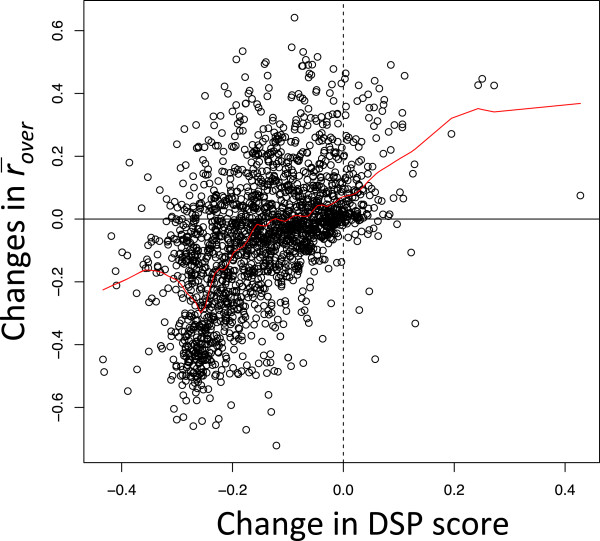
**Correlation of the DSP score and consistency with COG.** Each circle represents a pair of ortholog clusters that was one of the targets of the *merge* procedure. The red line was drawn by the *lowess* function of R with parameter *f* = 0.1.

Next, we examined the contribution of the DSP score to the refinement in the *merge* procedure. To quantify moderate agreement between two clustering results, we calculated the mean overlap ratio of corresponding domains (${\bar{r}}_{\mathrm{over}}$, see Methods for details). For each pair of adjacent clusters, we calculated the changes in the DSP score and the changes in ${\bar{r}}_{\mathrm{over}}$ after the merge for 2029 pairs of adjacent clusters and examined the correlation between them (Figure 6). We observed a positive correlation between them (Pearson’s correlation coefficient *r* = 0.51, *P* value of <1E-15). This observation supports an assumption that the DSP score is able to quantify the quality of domain-level ortholog classification in terms of consistency using the COG database as a reference. We drew a LOWESS curve to reveal the details of the relationship between the score changes and ${\bar{r}}_{\mathrm{over}}$ changes. When the score changes were positive, ${\bar{r}}_{\mathrm{over}}$ changes were mostly positive (128 pairs in positive and 22 in negative). Thus, we could safely merge clusters if the resulting score change was positive. In contrast, when the score changes were negative, ${\bar{r}}_{\mathrm{over}}$ changes varied, spanning positive (639 pairs) and negative (1185 pairs), meaning that some cluster pairs that should be merged may show negative score changes after the merge. In fact, the LOWESS curve demonstrated that when the score changes were small negative values, ${\bar{r}}_{\mathrm{over}}$ changes were slightly positive on average (for score changes between −0.05 and 0; the mean ${\bar{r}}_{\mathrm{over}}$ change was 0.06), suggesting that the threshold of the DSP score change for merging adjacent clusters should be a negative value rather than zero. This was desirable for avoiding the over-splitting problem because in this case, a domain split was introduced only when the splitting caused a sufficient score gain. On the basis of Figure 6, we used −0.05 as the threshold for the DSP score change to decide merges.

### Practical application of the refinement procedures

To demonstrate the utility of our method in a more practical situation, we applied the method to the FAMILY dataset that covers the diversity of currently sequenced microbial genomes, in addition to the COG03 dataset. We here used the TIGRFAMs database instead of the COG database to evaluate the clustering result. TIGRFAMs is a database containing the profile hidden Markov models (HMMs) constructed from manually curated multiple alignments of functionally equivalent protein families (equivalogs) [26] with “trusted cutoff” information for searching sequences with HMM using the HMMER program [27]. Thus, TIGRFAMs can be used to classify any set of protein sequences using the HMMER program. In addition, equivalogs defined in TIGRFAMs are a suitable reference classification for evaluating our ortholog classification, in that the main aim of the ortholog classification is to infer gene functions.

**Figure 7:**
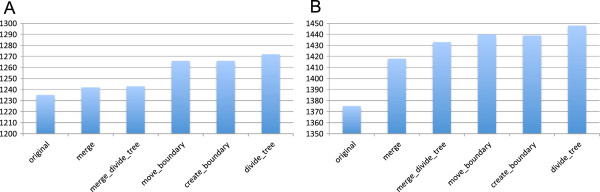
**Consistencies of the resulting clusters with TIGRFAMs models. (A)** COG03 dataset and **(B)** FAMILY210 dataset. Vertical axes represent the numbers of one-to-one ortholog relationships.

We applied our method (DomClust and DomRefine) to the COG03 and FAMILY datasets to classify genes and evaluated the resulting clusters using the TIGRFAMs database as a reference. As in the previous section, we considered the number of one-to-one corresponding cluster pairs against TIGRFAMs (*N*_*TIGR*_^*1to1*^) as a measure of consistency between two classifications. We examined the changes in *N*_*TIGR*_^*1to1*^ during the DomRefine procedure (Figure 7A, B) and again observed gradual increases during the DomRefine procedures in both the COG03 and the FAMILY210 datasets. In total, *N*_*TIGR*_^*1to1*^ was increased from 1235 to 1272 for the COG03 dataset and from 1375 to 1448 for the FAMILY210 dataset (Table 2).

**Table 2:** **Number of consistent clusters with TIGRFAMs models (*N***_***TIGR***_^***1to1***^**)**

**COG03 dataset**		**FAMILY210 dataset**	
**Method**	***N***_***TIGR***_^***1to1***^	**Method**	***N***_***TIGR***_^***1to1***^
COG	1107	eggNOG	1149
DomClust	1235 (1.12)	DomClust	1375 (1.20)
DomRefine	1272 (1.15)	DomRefine	1448 (1.26)
TIGRFAMs*	3576	TIGRFAMs*	3924

The ratio of *N*_*TIGR*_^*1to1*^ to COG or eggNOG is shown in parenthesis. *Number of TIGRFAMs models with hits in the corresponding dataset, which is the possible maximum number of *N*_*TIGR*_^*1to1*^.

However, some differences were observed between the results of this test (Figure 7A) and that of the previous test (Figure 5B), where the same COG03 dataset was used as a classification target, but COG instead of TIGRFAMs was used as the reference database. In particular, *N*_*TIGR*_^*1to1*^ was increased by the *divide_tree* procedure (Figure 7A), whereas *N*_*COG*_^*1to1*^ was decreased in the previous test (Figure 5B). In addition, *N*_*TIGR*_^*1to1*^ was less increased in the merge and *merge_divide_tree* steps, but more increased in the *move_boundary* step. Changes in the number of one-to-one ortholog relationships, illustrated in Figure 7, were analyzed in more detail by decomposing the change into gains and losses of one-to-one relationships (Additional file 1: Figure S2). Although occasionally a one-to-one relationship can be lost during the procedure, the gain of new relations significantly (*P* < 0.05 by binomial test) exceeds the losses in total and in most steps that have sufficient numbers of modifications (Additional file 1: Figure S1).

To compare the classification performance, we also evaluated the COG and eggNOG classifications in terms of the agreement with the TIGRFAMs models (*N*_*TIGR*_^*1to1*^). For the COG03 dataset, *N*_*TIGR*_^*1to1*^ of the original COG classification was 1107, whereas for the FAMILY210 dataset, *N*_*TIGR*_^*1to1*^ of the eggNOG classification was 1149 (Table 2). Both these values were even lower than those of the DomClust classification before refinement (1235 and 1375, respectively; Table 2). Thus, our DomClust/DomRefine classifications showed better agreement than the COG/eggNOG classifications when evaluated on the basis of the agreement with the TIGRFAMs classification.

To examine the inclusion relationships between corresponding ortholog groups in different ortholog classification systems, including DomClust/DomRefine, COG/eggNOG, and TIGRFAMs groups, we considered three additional concepts, equivalent, supergroup and subgroup that were introduced in our previous work [28] (Additional file 1: Table S1). The inclusion relationships among them tend to be COG > DomClust/DomRefine > TIGRFAMs > NOG, where A > B indicates that clusters in A tend to be supergroups of clusters in B. Note that a TIGRFAMs group can be a subgroup of a real orthologous group because of a strict trusted cutoff value, but the evaluation measure *N*_*TIGR*_^*1to1*^ is effective even in such a case, provided that there is a one-to-one relationship between the TIGRFAMs group and the corresponding target group.

### Examples of the resulting ortholog clusters

**Figure 8:**
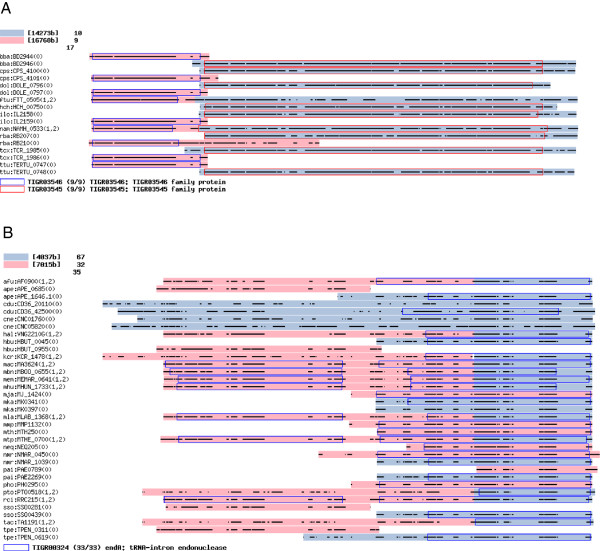
**Examples of the resulting ortholog clusters.** Examples of ortholog clusters obtained by DomRefine applied to the FAMILY210 dataset. **(A)** Clusters including genes split in the DomRefine result but not in eggNOG. **(B)** Clusters including genes with tandemly repeated domains. In these figures, coloring of each residue according to the conservation rate is disabled in order to simplify the representation.

On the basis of the resulting number of clusters for FAMILY210 (Table 1), the DomRefine result included a larger number of split clusters than eggNOG (10942 against 2333). We here focused on the genes split in the DomRefine result but not in eggNOG. Figure 8A presents an example of the clusters containing such genes, where two adjacent clusters corresponded to TIGRFAMs domains TIGR03546 and TIGR03545, respectively, both of which were functionally uncharacterized protein families. Although DomClust split a fused gene, nam:NAMH_0533 (*Nautilia profundicola*), into two domains, it failed to split another plausible fused gene, ftu:FTT_0505 (*Francisella tularensis*) (Additional file 1: Figure S3). However, DomRefine corrected the classification (Figure 8A). When the members of the clusters were compared to eggNOG, they overlapped three NOG clusters: NOG12793 (*N* = 6473), NOG44136 (*N* = 7), and NOG145366 (*N* = 2), where *N* indicates the cluster sizes in the FAMILY210 dataset. eggNOG did not split the two plausible fused genes, ftu:FTT_0505 and nam:NAM_0533; it assigned ftu:FTT_0505 to NOG145366 and nam:NAMH_0533 to NOG12793. As a result, proteins with the same TIGRFAMs hits were separated into different clusters. In contrast, NOG12793 was the largest eggNOG cluster containing proteins with many different TIGRFAMs hits (97 families), indicating that it is too large in terms of grouping corresponding genes among organisms.

Figure 8B presents another example, where the proteins had hits to TIGR00324 (*endA*: tRNA intron endonuclease). Here genes of FAMILY210 were extracted to demonstrate the subset of the alignment (see Additional file 1: Figure S4 for the full alignment of the FAMILY dataset). Of 35 proteins, 12 had two domains both of which correspond to TIGR00324, whereas in several species, these domains are coded as two separate genes. Some other species, such as *Methanocaldococcus jannaschii*, contain only one gene consisting of one domain (mja:MJ_1424). It is known that the two tandemly repeated domains, N-terminal repeat (NR) and C-terminal repeat (CR), have distinct functional roles and were suggested to have arisen by gene duplication and subfunctionalization [29]. Thus, it is reasonable to cluster these homologous domains into two distinct ortholog groups. When we created a phylogenetic tree using both the domains, we discovered distinct clusters corresponding to NR and CR. DomClust successfully clustered these domains except for two genes (Additional file 1: Figure S5), but DomRefine failed to refine these, in that the boundary modification reduced the agreement with TIGRFAMs hits (Figure 8B). One reason for this failure could be that the presence of tandemly repeated domains confounded the alignment, and DomRefine based on an incorrect alignment may fail to refine the domain boundary. In fact, in this case, single-domain proteins of *Nitrosopumilus maritimus*, nmr:NMAR_0450 and nmr:NMAR_1039, which were assigned to the NR and CR clusters, respectively, were both located in the C-terminal half in the alignment. Another problem affecting the alignment was the presence of unconserved sequences in the N-terminal regions of eukaryotic genes, such as cdu:CD36_42500 (*Candida dubliniensis*). In domain inferences of DomClust and DomRefine, these regions are treated as C-terminal groups (colored in light blue). Influenced by such an unconserved region, regions such as nmr:NMAR_0450 are prevented from being aligned to the N-terminal region and are consequently aligned to the C-terminal region.

## Discussion

In this study, we developed a method, DomRefine, to improve domain-level ortholog classification and applied the method to refine the ortholog classification created by our previous program, DomClust, using the proteome sets extracted from the COG and MBGD databases. We demonstrated that our method was able to achieve improvements when we evaluated the results on the basis of COG and TIGRFAMs, which are the manually curated reference databases. Although COG and TIGRFAMs clusters have different characteristics (as discussed below), DomClust clusters became more consistent with both COG and TIGRFAMs after the *merge* procedure of DomRefine (Figures 5 and 7), suggesting that the over-splitting problem in orthologous domains mentioned in the Background section were alleviated.

The TIGRFAMs database consists of HMMs constructed from curated multiple sequence alignments and is designed mainly for detecting functionally equivalent homologous proteins (equivalogs) among prokaryotic genomes [26]. Therefore, validating the obtained orthologous domains by TIGRFAMs models is reasonable in that the main aim of the ortholog database among prokaryotic genomes is to infer protein functions. In addition to the TIGRFAMs database, we used the COG database, a manually curated ortholog database for microbial genomes, as the reference database. However, when the same classification results of the COG03 dataset were evaluated using the different reference databases, COG and TIGRFAMs, we discovered different tendencies between them (Figure 5B and Figure 7A). In particular, the agreement with COG decreased after the *divide_tree* procedure (Figure 5B), whereas that with TIGRFAMs increased (Figure 7A). This difference is probably caused by the known COG problem that a substantial fraction of COG groups contain non-orthologous (or out-paralogous) genes [30]; thus, division of groups using the *divide_tree* procedure such that paralogous genes are appropriately separated can reduce the consistency with the COG classification. Another difference is that the *move_boundary* procedure improved domain boundaries in terms of their correspondence with TIGRFAMs (Figure 7A), whereas it failed to improve them in terms of their correspondence with COG (Figure 5B). This was observed because TIGRFAMs is constructed from the HMMs of well-conserved and well-characterized protein families, whereas COG was originally constructed from a clustering result based on all-against-all similarities. Consequently, the *move_boundary* procedure modified the domain boundaries to improve the coverage of well-conserved domain boundaries defined in TIGRFAMs, but may not have improved the correspondence with COG boundaries. In either case, we consider TIGRFAMs as a better reference dataset than COG to evaluate orthologous domain classification.

The goal of this study was to construct a fully automated and reliable procedure to create ortholog database, a necessary resource in the era of huge amounts of genomic data. In this respect, the eggNOG database, which was constructed by computational extension of COG, is another ortholog database that covers the currently sequenced genomes and is periodically updated. However, eggNOG consists of two different types of ortholog groups, i.e., the extension of the original COGs and the remaining NOGs, because of the nature of its incremental updating procedure. COG-derived clusters tend to be larger, whereas the NOG clusters tend to be smaller (Additional file 1: Table S1). As a result of the mixture of the two different distributions, the cluster size distribution of eggNOG appears to be deviated from the power-law distribution, which has been observed in various types of protein clusters [25] (Figure 4B).

To compare the classification performance, we also evaluated the COG and eggNOG clusters in terms of the agreement with the TIGRFAMs models (*N*_*TIGR*_^*1to1*^) and discovered that our method showed better agreement than the COG/eggNOG classifications (Table 2). The original DomClust classification already showed better agreement than the COG classification partly because of the abovementioned problem that some COG groups contain non-orthologous genes. In the eggNOG classification, additional problems caused by its incremental updating procedure can magnify the difference. In fact, the increasing rate of *N*_*TIGR*_^*1to1*^ from the eggNOG classification to the DomClust classification using the FAMILY210 dataset (20%) was higher than that from the COG classification to the DomClust classification (12%) (Table 2). The increasing rates were further increased when the COG/eggNOG classifications were compared to the classifications after refinement (15% and 26%, respectively; Table 2).

One of the problems with incremental updating in the eggNOG classification is that a new domain split appears to be rarely introduced during the NOG classification in contrast to the original COG classification (Table 1). Our DomClust/DomRefine procedure identified a substantial number of clusters that are not defined in COG, where domain splitting was needed for valid ortholog classification, as in the examples illustrated in Figure 8A. As illustrated in Figure 1A, a clustering method without domain splitting generally tends to create clusters with smaller sizes than that with domain splitting when fused proteins are included in the dataset. This may partly explain the smaller size distribution of the NOG clusters observed in Figure 4B.

Although numerous methods have been developed for identifying orthologs, few methods have focused on classification at the sub-gene level. Our method splits proteins into domains in the course of clustering with the aim of detecting the correct grouping of proteins (Figure 1A). The resulting splits of proteins suggest domain fusion/fission events in evolutionary history, which may result in functional divergence among orthologous proteins. In this sense, domain-level ortholog classification provides a valuable source for evolutionary analysis.

Theoretically, our system is applicable to eukaryotic protein classification. However, given the abundance of complex multidomain architectures among eukaryotic proteins and the frequent differences in domain composition among apparent orthologs [31,32], domain-level clustering of eukaryotic proteomes is more challenging than prokaryotic proteomes. In particular, as described in the Results section, a tandem repeat of homologous domains within a protein, which is quite common in eukaryotic proteins, may confound the multiple alignment, possibly leading to a failure of DomRefine to refine domain boundaries. As far as we tested, handling of tandemly duplicated domains seems to be more or less a common problem in existing alignment programs, although Clustal Omega used in this study demonstrated a relatively better performance with respect to this point. Thus, a special procedure may be required to handle such tandem repeats correctly as a pre- or postprocessing step of an alignment program unless improved versions of the alignment programs are available.

Although the current DomRefine pipeline requires much larger computational time than that required by DomClust, the parallelization technique enables the execution of the pipeline in a feasible time (Additional file 1: Table S2). Of the required time, the calculation of the DSP score comprises only a small fraction, and most of the computational time is spent performing multiple alignments. This bottleneck is caused by the repeated calculation of multiple alignments for the same set of sequences and could be partly solved by reusing the multiple alignment information. It is notable that the obtained multiple alignment information will be a useful resource not only for the DomRefine pipeline but also for various other applications. Therefore, it is worth computing and storing this information for general use.

## Conclusions

We developed a method for improving domain-level ortholog classification on the basis of the optimization of a score and demonstrated the effectiveness of the method using the manually curated reference databases. For this purpose, we designed a score for evaluating ortholog clusters at the domain level on multiple alignments and demonstrated that the method contributes to the improvement of the clusters. This method will enhance the reliability of ortholog databases and thereby contribute to comparative analyses using them.

## Methods

### Definition of the DSP score

The DSP score is calculated on the basis of multiple alignments. The score evaluates the consistency of domain-level ortholog clusters and multiple alignments. The basic idea is the sum-of-pairs score of a multiple alignment, which is a standard measure of evaluation of protein sequence alignment [33]. The unique idea of our score is that the calculation of the sum of pairs is restricted to specific domains, and that inconsistencies in the domain boundary positions are treated as gaps. Consider the alignment in the form of matrix *A* = (*a*_*ij*_), *i* = 1, ..., *N*_*seq*_ , *j* = 1, …, *N*_*pos*_, where *a*_*ij*_ represents an amino acid or a gap, *N*_*seq*_ is the number of sequences, and *N*_*pos*_ is the number of positions in the alignment. The positions of a domain on the amino acid sequences are also defined in the form of matrix *D* = (*d*_*ij*_) of the same size of *A*, where *d*_*ij*_ = 1 if *a*_*ij*_ is within the domain or otherwise *d*_*ij*_ = 0. The DSP score of domain *D* in multiple alignment *A* is given by

$$\begin{array}{l} S_{A}\left(D\right)=\sum_{i<i^{\prime }}^{N_{\mathrm{seq}}}[\sum_{j=1}^{N_{\mathrm{pos}}}\left\{s_{\mathrm{dom}}\left(a_{\mathrm{ij}},a_{i^{\prime }j},d_{ij},d_{i^{\prime }j}\right)\right\}\\ \;-n_{G_{\mathrm{open}}}\left(a_{i}.,a_{i^{\prime }.},d_{i.},d_{i^{\prime }.}\right)G_{\mathrm{open}}], \end{array}$$

where *G*_*open*_ is the gap-opening penalty. $n_{G_{\mathrm{open}}}\left(a_{i}.,a_{i^{\prime }.},d_{i.},d_{i^{\prime }.}\right)$ is the number of open gaps between the *i*-th sequence and *i*′*-*th sequence, where the open gaps are counted in the regions of *d*_*ij*_=1 and *d*_*i'j*_ = 1, and the mismatches of the domain terminal positions are counted as open gaps. *s*_*dom*_ is a function similar to a commonly used score matrix, but it returns a value depending on the domain as follows:

$$s_{\mathrm{dom}}\left(a,a^{\prime },d,d^{\prime }\right)=\left\{\begin{array}{c} s_{\mathrm{mat}}\left(a,a^{\prime }\right),\;\mathrm{if}\;b\left(a\right)d=1\;\mathrm{and}b\left(a^{\prime }\right)d^{\prime }=1\\ G_{\mathrm{ext}},\;\mathrm{else}\;\mathrm{if}\;b\left(a\right)d=1\;\mathrm{or}\;b\left(a^{\prime }\right)d^{\prime }=1\\ \;0,\;\mathrm{else}\;\mathrm{if}\;b\left(a\right)d=0\;\mathrm{and}\;b\left(a^{\prime }\right)d^{\prime }=0 \end{array}\right),$$

where *s*_*mat*_ is a commonly used score matrix such as the BLOSUM score matrices, *G*_*ext*_ is the gap extension penalty, and *b*(*a*) = 1 if *a* represents an amino acid or otherwise *b*(*a*) = 0. Therefore, a higher DSP score is obtained when the domain organization is such that sequence regions similar to each other (i.e., aligned with a positive score) belong to the same domain and sequence regions dissimilar to each other (i.e., aligned with a negative score) belong to different domains, because the DSP score counts similarity scores only between sequences belonging to the same domain. If the domain boundaries are not consistent with each other in the multiple alignment, they are penalized as external gaps, decreasing the score. Thus, an increase in the DSP score denotes that the domain boundaries are more consistent with each other in multiple alignment and/or the sequences belonging to the same domain produce a higher sum-of-pairs score. To normalize the DSP score with respect to the number of sequences and sequence lengths, we divide the DSP scores or the differences in the DSP scores by *N*_*seq*_ and *N*_*aa*_, where *N*_*aa*_ is the total number of amino acids included in the alignment.

### The *merge* procedure

In the *merge* procedure, all the split proteins in the dataset are re-examined in multiple alignments. Consider a pair of clusters that share at least one common protein whose sub-sequences are members of each cluster. We define two clusters as adjacent if they have a shared protein whose sub-sequences in each cluster are adjacent to each other in the shared protein sequence. To determine whether a pair of adjacent clusters should be merged, the DSP scores are evaluated before and after the merge. First, the score is calculated for each of the clusters before the merge. Then, the clusters are merged by canceling the split between the clusters. The clusters are to be merged under the condition of the normalized score change $\left(S^{\prime }-S\right)/\left(N_{\mathrm{seq}}N_{\mathrm{aa}}\right)>S_{\delta }$, where *S* and *S′* are the scores before and after the merge, respectively, and *S*_δ_ is a threshold for the merge. Following the examination of adjacent cluster pairs, all the pairs to be merged are merged at once.

### The *merge_divide_tree* procedure

The *merge_divide_tree* procedure temporarily merges a pair of adjacent clusters and then divides them into two groups as a split of a phylogenetic tree. Because this procedure is preceded by the *merge* procedure, we assume that clusters that should be merged are already merged.

A motivating example of this procedure is as follows: suppose there are two domains A and B. Some proteins have both domains (domain organization A + B) and the others have only domain A (domain organization A). In this case, we may want to classify these proteins into two groups corresponding to the two domain organizations, A + B and A, instead of the original domain-level classification, A and B. The *merge_divide_tree* procedure adopts the modified classification only when the resulting subgroups are consistent with the gene phylogeny, i.e., when they correspond to a split of the gene tree, as well as when the resulting DSP score becomes higher than before.

More precisely, this procedure re-defines the two groups on a phylogenetic tree as follows. If a root of the tree is determined, two subgroups are produced. The initial domain patterns are compared between the newly defined subgroups, and the difference is quantified as follows:

$$\begin{array}{l} t_{\mathrm{diff}}\left(G_{1},G_{2},t_{1},t_{2}\right)=\left|\left|g_{1}\;\cap t_{1}\right|+\left|g_{2}\;\cap^{\;}t_{2}\right|-\left|g_{1}\;\cap \;t_{2}\right|-\left|g_{2}\;\cap t_{1}\right|\right|\\ \;+\left|\left|g_{12\;}\cap^{\;}t_{1}\right|-\left|g_{12}\;\cap^{\;}t_{2}\right|\right|, \end{array}$$

where *G*_1_ and *G*_2_ represent initial clusters, *t*_1_ and *t*_2_ represent newly defined subgroups, *g*_12_ is the set of genes in both *G*_1_ and *G*_2_, *g*_1_ is the set of genes in *G*_1_ but not in *G*_2_, and *g*_2_ is the set of genes in *G*_2_ but not in *G*_1_. We calculated *t*_*diff*_ for all candidate roots and selected the root showing the largest *t*_*diff*_. If several candidate roots show the same value of *t*_*diff*_, the root with the longest edge among them is selected. Finally, the DSP score change was calculated comparing the original and resulting states, and the modification was executed only when the score increases.

### The *move_boundary* and *create_boundary* procedures

The *move_boundary* procedure moves the set of domain boundaries between two adjacent clusters at the same time, keeping them in the same column on the multiple alignment. By moving the position from the N terminus to the C terminus on the multiple alignment, the position showing the highest score is selected. If the best score is higher than the score of the initial state, the move of the boundaries is retained.

The *create_boundary* procedure creates a new boundary on candidate sequences, which are not split into domains in the initial state. Following the examination of all the protein sequences without splits, if the set of newly introduced splits increases the DSP score, boundary creation is applied.

### The *divide_tree* procedure

The *divide_tree* procedure checks whether the resulting clusters contain paralogous genes using a species overlap criterion that is used in DomClust as well as several tree-based ortholog classification methods. For this purpose, using FastTree, we created phylogenetic trees on the basis of multiple alignments produced by Clustal Omega. Although the obtained tree is unrooted, the root is placed on one of the edges so that the height of the resulting rooted tree is minimized. Division of a cluster into subgroups is determined by a species overlap rule as follows: $|S_{\mathrm{sp}}\left(t_{1}\right)\cap S_{\mathrm{sp}}\left(t_{2}\right)\left|/\right|S_{\mathrm{sp}}\left(t_{1}\right)\cup S_{\mathrm{sp}}\left(t_{2}\right)|\geq R_{\mathrm{sp}}$, where *t*_1_ and *t*_2_ represent candidate subgroups of the phylogenetic tree, *R*_*sp*_ is a threshold, and *S*_*sp*_(*t*_*i*_) represents the set of species included in *t*_*i*_.

### Dataset

The 2002 version of the COG database (COG02) contains genes from 43 species in 3307 clusters. We excluded ortholog groups comprising genes of fewer than three phylogenetically distinct organisms, retaining 3192 clusters, as described previously [20]. The 2003 version of the COG database (COG03) contains genes from 66 species in 4873 clusters [12]. Using the same filter applied to COG02, the number of clusters was reduced to 4814. DomClust was executed using the following parameters: *ao* (member overlap for merging adjacent clusters) of 0.8, *ai* (member overlap for absorbing adjacent small clusters) of 0.95, *V* (alignment coverage for domain split) of 0.6, and *C* (cutoff score for domain split) of 80. For the execution of the DomRefine pipeline, the following parameters were set: *G*_*open*_ of 10, *G*_*ext*_ of 0.5, *S*_*δ*_ of −0.05, and *R*_*sp*_ of 0.5, and BLOSUM45 was used as the score matrix *s*_*mat*_. In the tests to recover COG classification by DomClust, an additional parameter was used to specify a condition that at least three phylogenetically distinct organisms must be included in each cluster, as described previously [20].

The FAMILY dataset was created using the MBGD database [34]. Using NCBI taxonomy information, one representative genome was selected from each family. The resulting number of genomes was 309. COG and NOG clusters included in the eggNOG database v3.0 [35] were concatenated and designated as eggNOG in this study. To compare eggNOG classification with our classification based on the FAMILY dataset, we compared the list of genes between the FAMILY dataset and eggNOG v3.0 using NCBI taxonomy ID for organisms and locus ID for genes and extracted the intersection of these gene sets, obtaining a total of 587,463 genes from 210 organisms. Note that the eggNOG cluster sizes in the resulting FAMILY210 dataset were reduced from the original one because the species subset was extracted.

### Evaluation criteria

If overlapping fragments are observed between a COG cluster *C*_*i*_ and a DomClust cluster *D*_*j*_, whereas no overlapping fragments are observed between *C*_*i*_ and $D_{j^{\prime }}$ and between $C_{i^{\prime }}$ and *D*_*j*_ for any $j^{\prime }\neq j$ and $i^{\prime }\neq i$, then the relation of *C*_*i*_ and *D*_*j*_ is called a one-to-one relationship. When we have two clustering results, we can evaluate the consistency between them using the number of one-to-one relationships between them. To evaluate clustering results showing moderate agreement with the reference classification more appropriately than counting the number of one-to-one relationships, the agreement of clustering results was quantified as follows. The overlap ratio of fragment *c*∈*C*_*i*_ and fragment *d*∈*D*_*j*_ is calculated as *r*_*over*_ = |*c* ∩ *d*|/max(|*c*|, |*d*|). The mean overlap ratio $\;{\bar{r}}_{\mathrm{over}}$ is obtained by averaging  *r*_*over*_ for the overlapping fragments.

### Software

The core part of the pipeline that calculates the DSP score was implemented in the C language. Other parts of the pipeline are implemented in the Perl language. The programs were executed on Linux. DomClust [20] was used to obtain the initial clustering results. The pipeline accepts the DomClust default format, which includes the cluster members and the regions of the member domains. The DomRefine output is obtained in the same format as the input. Clustal Omega [36] was used to create multiple alignment with *auto* option. FastTree [37] was used to create a phylogenetic tree based on the multiple alignment produced by Clustal Omega. For visualizing domain-level clustering results on multiple alignments, we developed a visualization tool using Perl and the GD library (http://search.cpan.org/dist/GD/). The tool colors the amino acid residues according to the conservation rate *p*_*cons*_ in the multiple alignment: red for *p*_*cons*_ ≥ 70%, yellow for 70% > *p*_*cons*_ ≥ 50%, and cyan for 50% > *p*_*cons*_ ≥ 30%. The scatter plot was created using R (http://www.r-project.org/). A significance test of the results obtained by the binomial test was performed using the *binom.test* function of R considering gains and losses as successes and failures in trials, respectively. TIGRFAMs release 13.0 [26] was used as protein models. For searching the protein sequences using the protein models, HMMER3 [27] was used with the “trusted cutoff” of each model. DomRefine including the visualization tool can be downloaded from the following link: http://mbgd.genome.ad.jp/domrefine/.

## Abbreviations

DSP score: Domain-specific sum-of-pairs score; MBGD: Microbial Genome Database for Comparative Analysis; COGs: Clusters of Orthologous Groups; NOGs: Non-supervised orthologous groups; HGT: Horizontal gene transfer.

## Competing interests

The authors declare that they have no competing interests.

## Authors’ contributions

HC designed the study, performed the analysis, and drafted the manuscript. IU conceived of the study, participated in its design, and helped to draft the manuscript. All authors read and approved the final manuscript.

## Supplementary Material

Additional file 1 Supplementary figures and tables. Click here for file
