# Supplementary material for: Improvement of domain-level ortholog clustering by optimizing domain-specific sum-of-pairs score
Source: BMC Bioinformatics. 2014 May 18;15:148. doi: 10.1186/1471-2105-15-148 (PMC4035852; doi:10.1186/1471-2105-15-148)
Supplement: Additional file 1 — Supplementary figures and tables. [file 1471-2105-15-148-S1.pdf]

Figure S1. Cluster size distributions of domain-level ortholog clusters. Clustering results for COG03 dataset (A) and FAMILY210 dataset (B). (A) This analysis is same as that for Figure 4A, except that DomClust was here executed with options which allow generation of ortholog groups with less than three members (domclust -n1 -ne1). A line is fitted to the DomClust distribution by linear regression ($\log_{10} y = -1.789 \log_{10} x + 4.240$, R^2=0.90). (B) This analysis is same as that for Figure 4B, but the line was fitted to the eggNOG distribution ($\log_{10} y = -1.228 \log_{10} x + 3.486$, R^2=0.85) and NOG distribution ($\log_{10} y = -1.838 \log_{10} x + 3.885$, R^2=0.71).

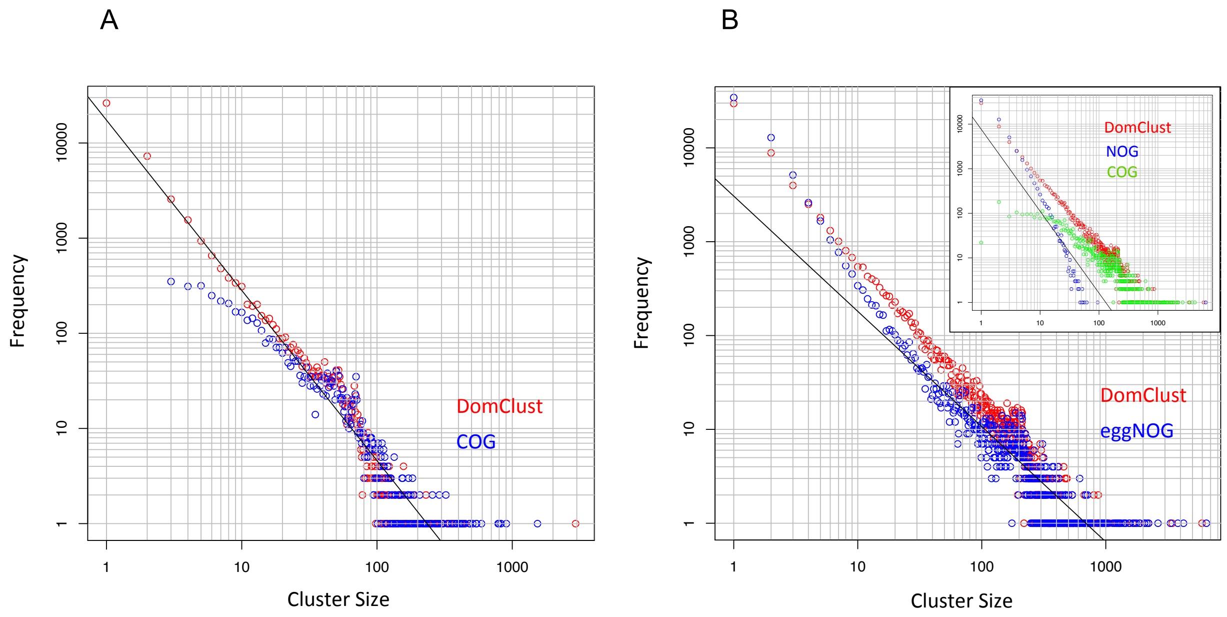

Figure S2. Gains and losses of one-to-one relationships between ortholog clusters and TIGRFAMs models. (A) COG02 dataset, (B) COG03 dataset and (C) FAMILY210 dataset. The blue bars represent gains of one-to-one relationships and the red represents losses. Significant differences of the gains and losses with $P < 0.05$ by binomial test are indicated by *.

A

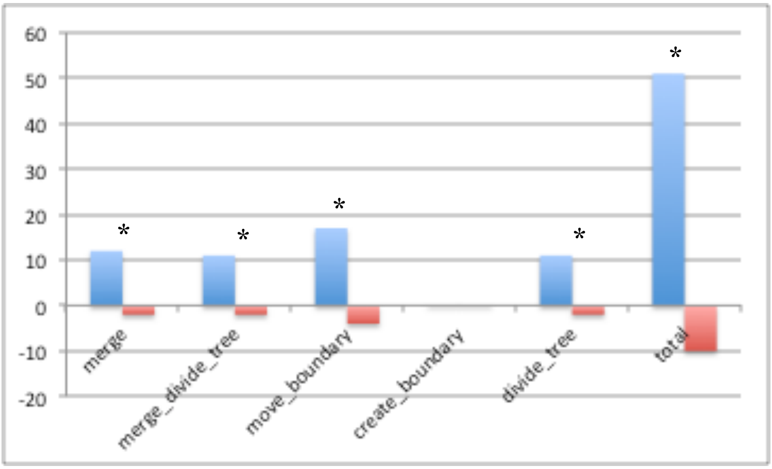

Total: 51 gains and 10 losses (*P* = 9.62E-8 by binomial test)

B

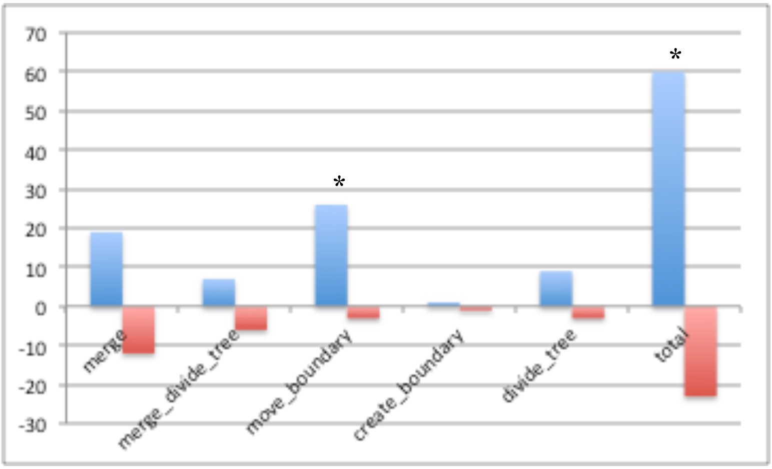

Total: 60 gains and 23 losses (*P* = 5.97E-5 by binomial test)

C

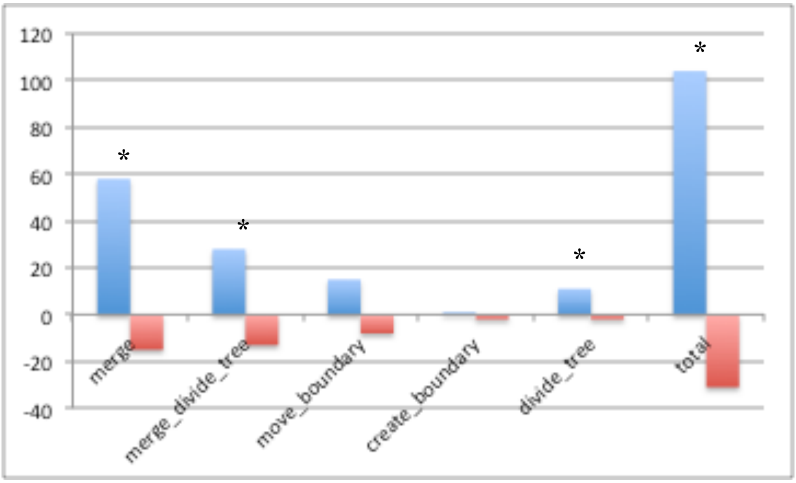

Total: 104 gains and 31 losses (P = 2.06E-10 by binomial test)

Figure S3. Examples of ortholog clusters for FAMILY210 dataset. The proteins contained in these examples are same as those in Figure 8A. Results of DomClust before applying DomRefine are shown.

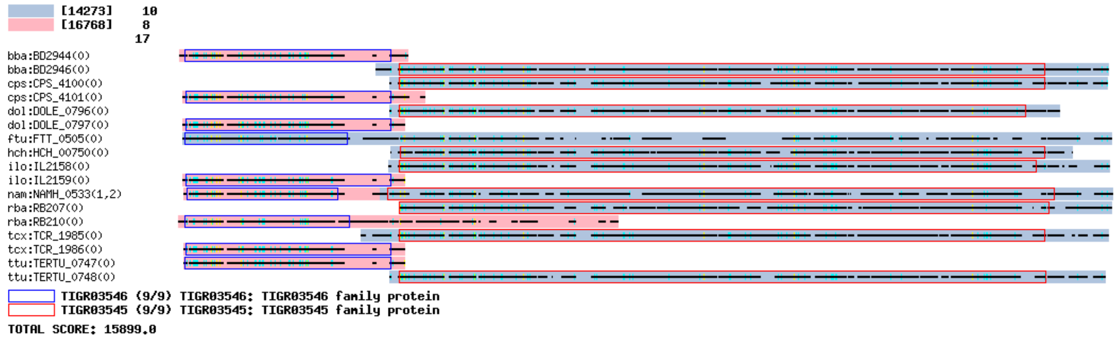

Figure S4. Examples of ortholog clusters. The clusters correspond to those in Figure 8B. When creating this figure, the organisms are not restricted to 210 organisms of FAMILY210 dataset, considering the 309 organisms of FAMILY dataset.

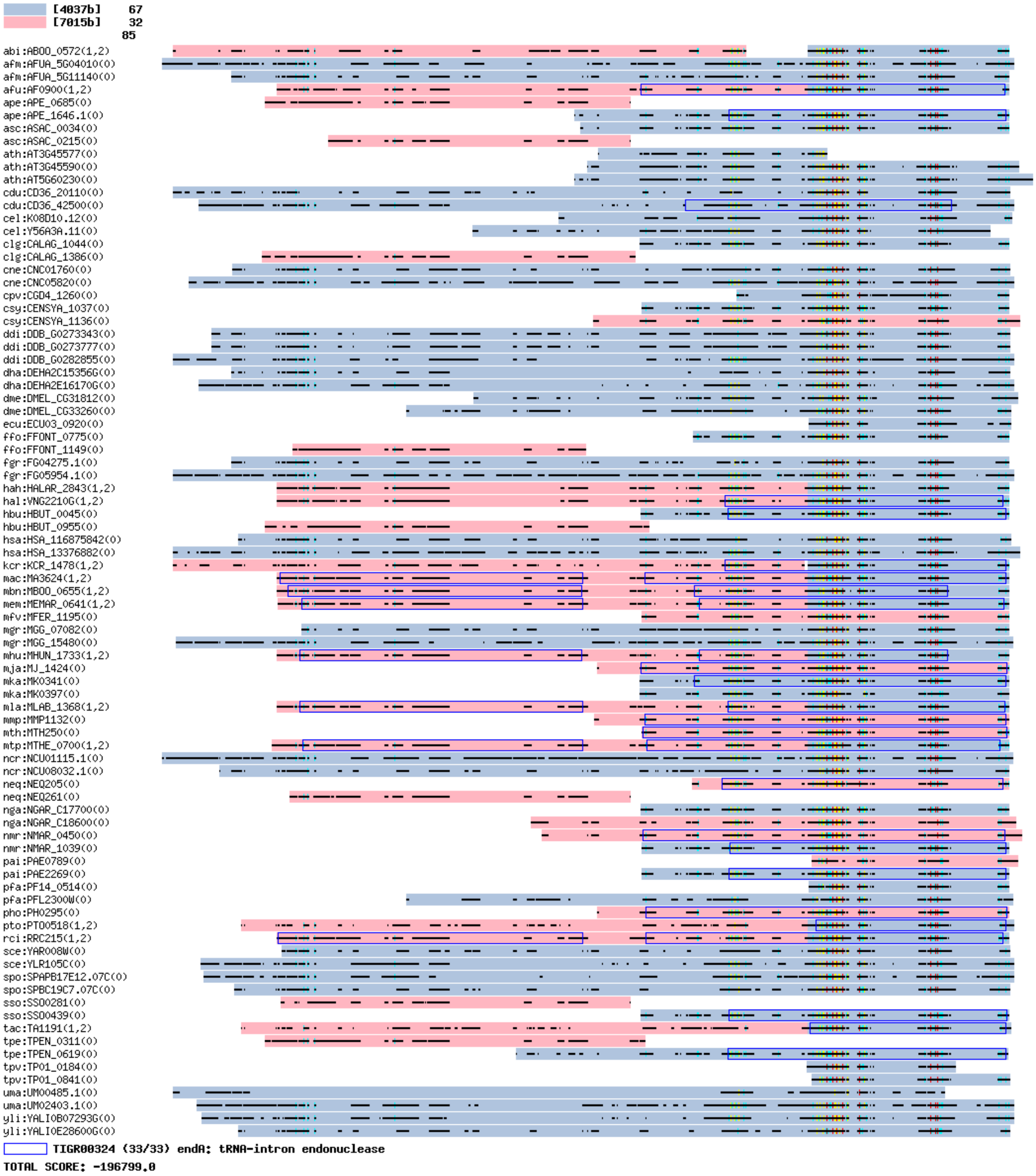

Figure S5. Examples of ortholog clusters for FAMILY210 dataset. The proteins contained in these examples are same as those in Figure 8B. Results of DomClust before applying DomRefine are shown. (A) The protein sequences are aligned by Clustal Omega. Domains are colored in pink or light blue acocording to the DomClust results. (B) After the proteins are split into domains, those domains are aligned by Clustal Omega (domain by domain), and the phylogenetic tree of them are created by FastTree. In the tree, we found distinct clusters corresponding to N-terminal repeat (NR) and C-terminal repeat (CR). The leaves colored in red and blue correspond to the DomClust cluster colored in pink and light blue in (A), respectively. DomClust successfully clustered the domains except two genes.

A

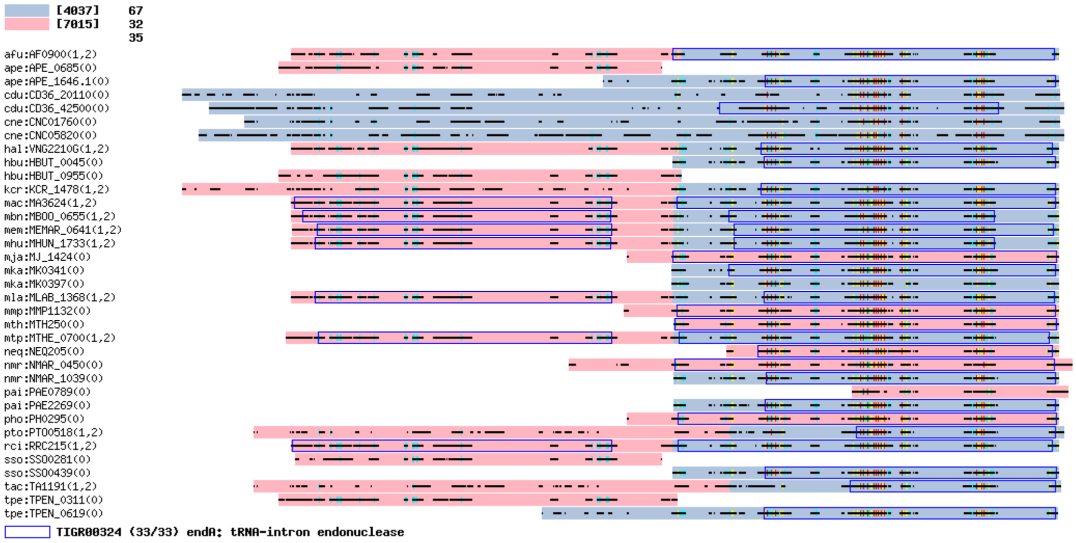

B

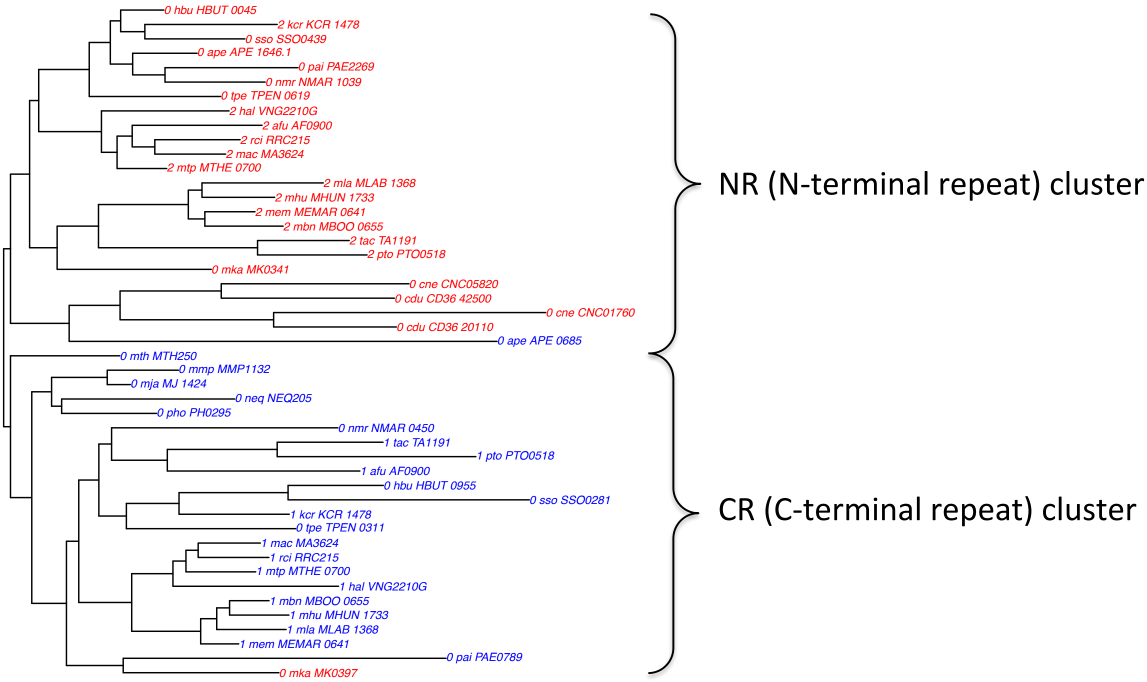

Table S1. The number of reference clusters being equivalent, subgroup and supergroup of obtained clusters.

COG03 dataset					
**Clusters**	**Reference**	N_{ref}^{equiv}	N_{ref}^{sub}	N_{ref}^{super}	N_{clust}
**COG**	**TIGRFAMs**	1271	1678	55	4814
**DomClust**	**TIGRFAMs**	1364	1342	102	7503
**DomRefine**	**TIGRFAMs**	1386	1389	106	7308
**DomRefine**	**COG**	3618	359	779	7308

FAMILY210 dataset					
**Clusters**	**Reference**	N_{ref}^{equiv}	N_{ref}^{sub}	N_{ref}^{super}	N_{clust}
**eggNOG**	**TIGRFAMs**	1448	1828	587	64983
COG*	**TIGRFAMs**	1004	1721	84	4873
NOG*	**TIGRFAMs**	444	107	564	60110
**DomClust**	**TIGRFAMs**	1652	1524	306	60775
**DomRefine**	**TIGRFAMs**	1674	1674	308	57644
**DomRefine**	**eggNOG**	35542	26691	4806	57644
**DomRefine**	COG*	3763	735	1998	57644
**DomRefine**	NOG*	31779	25956	2808	57644

N_{ref}^{equiv}, N_{ref}^{sub} and N_{ref}^{super} represent the number of reference clusters that are equivalent, subgroup and supergroup of the cluster, respectively. N_{clust} is the total number of clusters obtained by each method. Let $C \wedge R$ denote a set of corresponding segment pairs between a cluster C and a reference cluster R. Here, we considered that a segment $s_c \in C$ corresponds to a reference segment $s_r \in R$ if $|s_c \cap s_r|/|s_r| \geq 0.9$. Let $p_c = |C \wedge R|/|C|$, $p_r = |C \wedge R|/|R|$ and $F = 2p_c p_r/(p_c+p_r)$. We defined R as being equivalent to C if $F \geq 0.7$; otherwise, R is a subgroup of C if $p_r \geq 0.7$ or a supergroup of C if $p_c \geq 0.7$.

Each raw represents the result of comparison between obtained clusters and reference clusters. If $N_{ref}^{sub} > N_{ref}^{super}$, then the obtained clusters tend to be larger than the reference clusters. If $N_{ref}^{sub} < N_{ref}^{super}$, then the obtained clusters tend to be smaller than the reference clusters.

*eggNOG clusters were divided into COG-derived clusters and NOG clusters.

Table S2. The execution time of DomClust and DomRefine for COG03 dataset.

Method	Time (minutes)
**DomClust**[a]	1.90
**DomRefine**[b]	352.48
Total*	16507.08 (100%)
Clustal Omega*	13353.60 (80.9%)
FastTree*	744.42 (4.5%)
DSP score*	212.56 (1.3%)
Others*	2196.50 (13.3%)

[a]DomClust was executed on a single core of Intel Xeron 2.7 GHz. The calculation of DomClust does not include the construction of all-against-all similarity data.

[b]DomRefine was executed on a parallel environment including a single core of Intel Xeon 2.7 GHz and 100 cores of Intel Xeron 2.8 GHz through a job management system based on Sun Grid Engine. The real time required to finish the computation on the environment was measured.

*In the case of DomRefine, the execution time measured on each core was totalized for all the processes (Total), or for a specific type of processes (Clustal Omega, FastTree, DSP score and Others).
